# Novel Mesoporous Organosilicas with Task Ionic Liquids: Properties and High Adsorption Performance for Pb(II)

**DOI:** 10.3390/molecules27041405

**Published:** 2022-02-18

**Authors:** Karolina Wieszczycka, Kinga Filipowiak, Patrycja Dudzinska, Marek Nowicki, Katarzyna Siwińska-Ciesielczyk, Teofil Jesionowski

**Affiliations:** 1 Faculty of Chemical Technology, Institute of Chemical Technology and Engineering, Poznan University of Technology, Berdychowo 4, 60-965 Poznan, Poland; kinga.m.filipowiak@doctorate.put.poznan.pl (K.F.); patrycja.dudzinska1997@gmail.com (P.D.); katarzyna.siwinska-ciesielczyk@put.poznan.pl (K.S.-C.); teofil.jesionowski@put.poznan.pl (T.J.); 2 Faculty of Materials Engineering and Technical Physics, Institute of Physics, Poznan University of Technology, Piotrowo 3, 60-965 Poznan, Poland; marek.nowicki@put.poznan.pl; 3 Center for Advanced Technology, Adam Mickiewicz University, Uniwersytetu Poznańskiego 10, 61-614 Poznan, Poland

**Keywords:** ionic liquid, adsorbent, functionalized silica, Pb(II) removal

## Abstract

Removal of toxic contaminants such as Pb(II) from waste solutions is environmentally requested. Therefore, in this paper, for potential novel sorbents, mesoporous ionic liquid-functionalized silicas were synthesized and tested for the removal of Pb(II) from aqueous solutions. The successful synthesis of the adsorbents was proved by nuclear magnetic resonance (^29^Si and ^13^C NMR), Fourier transform infrared spectroscopy (FTIR), and elemental analysis. The structural and textural properties were determined using scanning electron microscopy (SEM), X-ray diffraction (XRD), high-resolution transmission electron microscopy (TEM), and low-temperature N_2_ sorption, and the result showed that the applied procedure made it possible to obtain highly ordered particles with a two-dimensional mesostructure. The effects of several parameters including initial pH, contact time, adsorption temperature, and Pb(II) concentration were studied in detail and were discussed to evaluate the adsorption properties of the fabricated materials towards Pb(II). The obtained results confirmed a very high potential of the sorbents; however, the adsorption properties depend on the structure and amounts of the functional group onto fabricated materials. The sample ILS-Ox3-40 showed fast kinetics (equilibrium reached within 10 min) and capacity of 172 mg/g, and that makes it a promising sorbent for the cleanup of water contaminated by lead. It was also indicated that, regardless on structure of the tested materials, the Pb(II) removal was spontaneous and exothermic. The fabricated mesoporous silicas exhibited that they were easy to regenerate and had excellent reusability.

## 1. Introduction

Mesoporous silicas are currently the most promising nanostructured materials showing applicability in many areas. They have been considered an important class of materials used as catalysts or supports for catalysts [1], fillers of polymer and polymer-based materials (to improve mechanical, thermophysical, and electrical properties) [2,3,4,5,6], adsorbents of hazardous wastewaters’ pollutants [7,8,9], and as bioactive materials (drug delivery systems, biosensors, protein adsorbents) [10,11,12,13,14,15]. The indicated applications are mainly due to the structural properties of the material, especially the large internal surface area and pore volume. However, the adsorption of metals’ ions such as toxic Pb(II) requires the presence of groups that will support the structural properties, resulting in not only efficient but also selective adsorption [16,17,18,19,20,21,22]. The choice of functionalities relies upon the type of metal ions to be adsorbed, the pH of the solution, and the type of accompanying ions. For example, Pb(II) can be adsorbed by silica consisting of propylethylenediamine [23], 2,2′-(pentane-1,5-diylbis(oxy))dibenzaldehyde [24], norepinephrine [25], melamine-based dendrimer amines [26], and pyridylpyrazole-β-ketoenol [27] groups. An important class of ligands, with a higher affinity for hard and soft metals, is ionic liquid (IL) such as pyridinium derivatives, which are also very promising for Pb(II) removal both in liquid–liquid and liquid–solid systems [28,29,30,31]. The wide use of ionic liquids as extractants in the liquid–liquid system and as ionic liquids’ functionalities on a silica or polymer support as adsorbents results from their multi-functionality. Ionic liquid molecules mainly interact with anionic and neutral metals’ species, and their properties can also be closely aimed at a target metal species by introducing additional groups into the structure of cationic moiety. Examples of ionic liquid-based extractants are quaternary salts of 1-(pirydne-3- or -4-yl)undecan-1-one and their oxime and appropriate imine derivatives. In classical use, these compounds show very good extraction properties towards metals, selectivity, ease of regeneration, and stability under strongly acidic conditions [32,33,34]. In an adsorption system, the chloride salts were introduced into the silica and polymer matrix, thanks to which materials with above-average sorption properties towards cationic species of Cu(II), Zn(II), Cd(II), and anionic Cr(VI) were obtained [35,36,37,38]. The results obtained so far are promising, but the full potential of these types of materials has not yet been demonstrated including, in particular, a stable functionalization with a highly ordered mesoporous structure. Both of these features should make it possible to effectively process the purification of waste solutions from toxic metal impurities.

In view of the above, in the present study the potential of novel IL-functionalized silica adsorbents were explored. The research concerned the influence of the type and quantity of precursor functional groups on the composition, morphology, and textural properties of the fabricated materials and, thus, also on the adsorption properties towards Pb(II) ions.

## 2. Materials and Methods

### 2.1. Chemicals

Pluronic P-123 (poly(ethylene oxide)_20_-poly(propylene oxide)_70_-poly(ethylene oxide)_20_), tetraethyl orthosilicate (TEOS, 99.99%), and ethanol (absolute, EMPROVE^®^) used for the fabrication of the functionalized mesoporous silica particles were purchased from Sigma Aldrich (Darmstadt, Germany). The precursors of the ionic liquids’ groups (ILS, 1-[3-(trimethoxysilyl)propyl]-3-undecanoylpyridinium bromide, 1-[3-(trimethoxysilyl)propyl]-4-undecanoylpyridinium bromide, 3-[1-(hydroxyimine)undecyl]-1-[3-(trimethoxysilyl)propyl]pyridinium bromide, and 4-[1-(hydroxyimine)undecyl]-1-[3-(trimethoxysilyl)propyl]pyridinium bromide were synthesized by quaternization of 1-(pyridin-3-yl)undecan-1-one, 1-(pyridin-4-yl)undecan-1-one and their oximes (purities 98.8-99.1%) [39] with (3-bromopropyl)trimethoxysilane (≥97%, Sigma Aldrich, Darmstadt, Germany) using acetonitrile as diluent. The quaternization was conducted at 40 °C for 72 h and under argon atmosphere.

*1-[3-(trimethoxysilyl)propyl]-3-undecanoylpyridinium bromide* (**K3)** ^1^H-NMR (CDCl_3_) δ in ppm: 9.21 (s, 1H, H_py2_); 8.64 (d, 1H, H_py6_); 8.23 (d, 1H, H_py4_); 7.33 (t, 1H, H_py5_); 4.95 (t, CH_2_-Br); 3.28 (s, 9H, CH_3_), 3.17 (t, 2H, CH_2_); 2.21 (q, 2H, CH_2_); 1.66 (t, 2H, CH_2_); 1.56 (t, 2H, CH_2_); 1.4-1.21 (m, 16H, CH_2_); 1.13 (t, 2H, CH_2_Si); 0.90 (t, 3H, CH_3_); ^13^C NMR (CDCl3,) *δ* in ppm: 198.1 (C=O); 146.3 (C_py2_); 145.8 (C_py6_); 137.7 (C_py4_); 132.4 (C_py3_); 128.6 (C_py5_); 58.6 (CH_2_-N^+^); 52.9 (CH_3_-O); 41.9 CH_2_-C=O); 32.1 (CH_2_); 31.3 (CH_2_); 29.7 (CH_2_); 29.5 (CH_2_); 29.4 (CH_2_), 29.3 (CH_2_); 24.6 (CH_2_); 23.7 (CH_2_); 18.7 (CH_2_); 13.9 (CH_3_); 10.7 (CH_2_); 9.9 (CH_2_-Si).

*1-[3-(trimethoxysilyl)propyl]-4-undecanoylpyridinium bromide* (**K4)** ^1^H-NMR (CDCl_3_) δ in ppm: 8.86 (s, 1H, H_py2,6_); 8.02 (d, 1H, H_py3,5_); 4.92 (t, CH_2_-Br); 3.55 (s, 9H, CH_3_); 3.01 (t, 2H, CH_2_); 2.18 (q, 2H, CH_2_); 1.63 (t, 2H, CH_2_); 1.54 (t, 2H, CH_2_); 1.43-1.12 (m, 16H, CH_2_); 1.16 (t, 2H, CH_2_Si); 0.89 (t, 3H, CH_3_); ^13^C NMR (CDCl3,) *δ* in ppm: 199.6 (C=O); 149.4 (C_py2,6_); 121.6 (C_py3,5_); 58.1 (CH_2_-N^+^); 52.3 (CH_3_-O); 41.5 CH_2_-C=O); 32.4 (CH_2_); 31.2 (CH_2_), 29.5 (CH_2_); 29.4 (CH_2_); 29.3 (CH_2_); 29.2 (CH_2_); 24.7 (CH_2_); 23.7 (CH_2_); 18.4 (CH_2_); 13.6 (CH_3_); 11.1 (CH_2_); 9.1 (CH_2_-Si)

*3-[1-(hydroxyimine)undecyl]-1-[3-(trimethoxysilyl)propyl]pyridiniumbromide* (**Ox3)** ^1^H-NMR (CDCl_3_) δ in ppm: 8.96 (s, 1H, N-OH); 8.61 (d, 1H, H_py2_); 8.07 (d, 1H, H_py6_); 7.96 (d, 1H, H_py4_); 7.32 (t, 1H, H_py5_); 4.91 (t, CH_2_-Br); 3.35 (s, 9H, OCH_3_) 2.98 (t, 2H, CH_2_); 2.18 (q, 2H, CH_2_); 1.66 (t, 2H, CH_2_); 1.57 (t, 2H, CH_2_); 1.43-1.13 (m, 16H, CH_2_); 1.11 (t, 2H, CH_2_Si); 0.89 (t, 3H, CH_3_);^13^C NMR (CDCl_3_) δ in ppm: 159.1 (oxime C=N); 147.7 (C_py2_); 144.2 (C_py6_) 137.6 (C_py4_); 134.5 (C_py5_); 123.8 (C_py3_); 57.6 (CH_2_-N^+^); 50.7 (CH_3_-O); 50.1 CH_2_-C=N); 32.1 (CH_2_); 31.3 (CH_2_); 29.7 (CH_2_); 29.5 (CH_2_); 29.4 (CH_2_); 29.3 (CH_2_); 24.6 (CH_2_); 23.7 (CH_2_); 18.7 (CH_2_); 13.8 (CH_3_); 10.7 (CH_2_); 9.9 (CH_2_-Si).

*4-[1-(hydroxyimine)undecyl]-1-[3-(trimethoxysilyl)propyl]pyridiniumbromide* (**Ox4)** ^1^H-NMR (CDCl_3_) δ in ppm: 9.62 (s, 1H, OH); 8.74 (d, 1H, H_py2,6_); 7.81 (d, 1H, H_py3,5_); 4.89 (t, CH_2_-Br); 3.55 (s, 9H, CH_3_) 2.87 (t, 2H, CH_2_); 2.22 (q, 2H, CH_2_); 1.64 (t, 2H, CH_2_); 1.55 (t, 2H, CH_2_); 1.4-1.25 (m, 16H, CH_2_); 0.87 (t, 3H, CH_3_), 0.64 (t, 2H, CH_2_)^13^C NMR (CDCl_3_) δ in ppm: 158.7 (oxime C=N); 144.4 (C_py2,6_); 118.3 (C_py3,5_); 154.1 (C_py4_); 52.5 (CH_3_-O); 58.1 (CH_2_-N^+^); 50.5 CH_2_-C=N); 32.3 (CH_2_); 31.6 (CH_2_); 29.8 (CH_2_); 29.7 (CH_2_); 29.6 (CH_2_); 29.5 (CH_2_); 24.7 (CH_2_); 23.9 (CH_2_); 21.5 (CH_2_); 14.1 (CH_3_); 10.5 (CH_2_); 9.6 (CH_2_-Si).

Ultra-pure water (with resistivity of 18.2 MΩ cm) used in the synthesis and sorption tests was obtained by using an Arium Pro DI purification system (Sartorius, Göttingen, Germany). NaCl (99.99%), HCl (35%, p.a.), lead(II) nitrate (99.999%), Pb standard solution (AAS standard), and buffers were of analytical grade and were purchased from Merck KGaA (Darmstadt, Germany).

### 2.2. Procedures

#### 2.2.1. Organosilicas’ Fabrication

**Figure 1:**
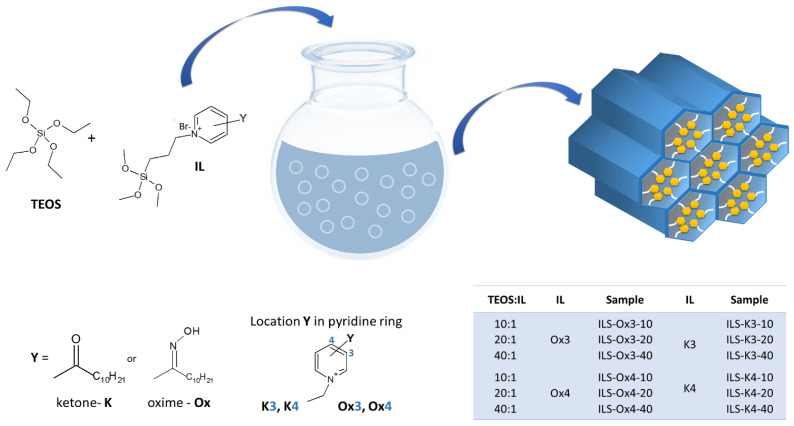
Scheme of sorbents’ synthesis.

The ionic liquid-functionalized silicas were synthesized through co-condensation of tetraethyl orthosilicate (TEOS) and the appropriate precursors of the ionic liquid functional groups (IL) (Figure 1). The molar ratio of TEOS to IL was 40, 20, and 10. In the first stage of the synthesis, 2.0 g of Pluronic P-123 was dissolved in 75.0 mL of aqueous solution of HCl (1.5 M). The mixture was stirred at 40 °C for 3 h. Then, to the dissolved Pluronic 123 mixture, TEOS (4.0 g), the specified amount of IL and 2 mL of ethanol were added at 40 °C under vigorous stirring. The heating was continued for 1 day and subsequently heated at 100 °C for 2 days. The resulting precipitate was filtered, followed by extraction of the Pluronic P-123 with the acidic ethanol solution for 24 h at 60 °C.

#### 2.2.2. Characterization of the Synthesized Organosilicas

FTIR spectra of the fabricated powders were collected using a Vertex 70 (Bruker, Ettlingen, Germany) spectrophotometer at room temperature in the 4000 to 400 cm^−1^ region at a resolution of 0.5 cm^−1^. The samples were analyzed after dispersing the samples in KBr pellets. Elemental analysis was carried out on a Vario ELIII Elementar analyzer (Elementar Analysensysteme GmbH, Langenselbold, German). Electron microscopic measurements were made with the FEI Quanta 250FEG scanning electron microscope. The analysis was carried out at an accelerating voltage of 20 kV and at high vacuum mode. Prior to imaging, the samples were covered with a 20-nm gold layer in a vacuum sputter. Transmission electron microscopy (TEM) investigation was carried out using a HT7700 microscope (Hitachi, Tokio, Japan), operating at an accelerating voltage of 120 kV and equipped with the 2k × 2k CCD camera (AMT XR41, Woburn, MA, USA). The sample was dispersed in ethanol and subjected to an ultrasonic bath. Samples were dropped on carbon-coated copper grids for the TEM observations. The low-temperature N_2_ sorption at −196 °C was applied to evaluate the textural properties, such as surface area (A_BET_), total pore volume (V_p_), and pore size (S_p_), of the fabricated materials. The physisorption analyzer ASAP 2020 (Micromeritics Instrument Co., Norcross, CA, USA) was utilized for this purpose. The Brunauer–Emmett–Teller (BET) model was used to calculate the surface area of the fabricated products based on adsorption data for relative pressure *(p*/*p_0_*) in the range of 0.05–0.30. The desorption isotherm was used to determine the pore size distribution and the total volume of pores based on the Barrett–Joyner–Halenda (BJH) method, using the Halsey equation. Before measurement, the fabricated materials were degassed at 120 °C for 4 h. Low-angle (2*θ* = 0.4–5.5°) X-ray diffraction patterns were recorded on a Panalytical Empyrean diffractometer (Malvern Panalytical, Malvern, UK) equipped with *θ*–*θ* Bragg Brentano geometry (Cu Kα wavelength, λ =1.5418 Å). Low-angle diffraction patterns were recorded using a zero-background silicon sample holder. The XPS analyses of the selected sorbents before and after the metals’ adsorption were carried out in a PHI VersaProbeII Scanning XPS system using monochromatic Al Kα (1486.6 eV) X-rays focused to a 100-µm spot. The photoelectron takeoff angle was 45°, and the pass energy in the analyzer was set to 117.50 eV for survey scans and 46.95 eV to obtain high-energy resolution spectra for the C 1s, N 1s, O 1s, Si 2p, and Br 3d regions.

#### 2.2.3. Pb(II) Sorption

Sorption experiments were carried out by employing the batch method by mixing 0.1 g of the fabricated materials with 100 mL of aqueous solutions of Pb(II). The sorption was studied using a temperature-controlled shaker (KS 4000 ic control, IKA) at different pH values (1–6), times (1–350 min), temperatures (23–45 °C), and at different Pb(II) concentrations (50–200 mg/L). The atomic absorption spectrometer (AAS—ContrAA 300, Analytik Jena, Jena, Germany) was used for the measurement of Pb(II) ion concentrations in the aqueous samples before and after sorption and desorption. A Mettler-Toledo pH-meter with a DG115-SC pH electrode was used for pH measurement. All sorption tests were replicated three times to obtain the mean value. The amount of adsorbed Pb(II) onto the fabricated products was calculated using Equation (1), where *C*_0_ and *C_t_* (mg/L) are concentrations of Pb(II) ions before and after adsorption at time *t*, *V* is volume of the aqueous phase used (L), and *m* is the mass of the adsorbent used (g).

(1) $$q=\frac{\left(C_{0}-C_{t}\right)\cdot V}{m}$$

## 3. Results

### 3.1. Characterization of Fabricated Materials

#### 3.1.1. Synthesis Confirmation

**Figure 2:**
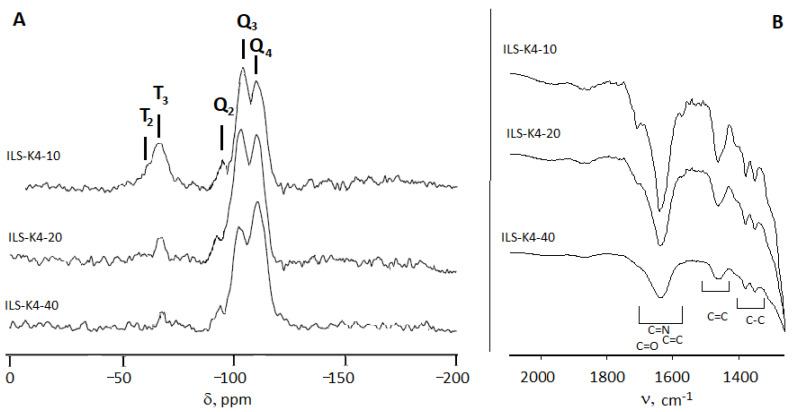
The ^29^Si MAS NMR (**A**) and FT-IR spectra (**B**) of materials’ series ILS-K4.

Confirmation of the functional group incorporation was made using the solid-state ^13^C and ^29^Si CP-MAS NMR spectra as well as XPS and elemental analysis. The ^29^Si MAS NMR analysis revealed the presence of three broad peaks at −92, −101, and −110 ppm assigned to Q_2_, Q_3_, and Q_4_ silica structural units (Si(OSi)_2_(OH)_2_), Si(OSi)_3_(OH), and Si(OSi)_4,_ respectively) (Figure 2a). These peaks were also accompanied by signals corresponding to the CH_2_-Si(OSi)_2_(OR) and CH_2_-Si(OSi)_3_ moieties (T_2_ and T_3_ at −66.7 ppm, respectively), which confirmed the presence of the silicon bonded to the propylpyridinium group. It was also observed that the presence of the silanol moiety closely correlated with the number of functional groups introduced into the silica structure. With the 40-fold excess of TEOS to ILS, the signal corresponding to T_2_ and Q_2_ was almost invisible and the signal Q_4_ dominated the signal Q_3_ (the Q_3_/Q_4_ ratio was 0.80, 0.78, 0.81, and 0.83 for ILS-Ox3-40, ILS-Ox4-40, ILS-K3-40, and ILS-K4-40, respectively). However, when the 10-fold excess of TEOS was used in the synthesis, the participation of functional groups in the formation of the silicate structure was more significant (the Q_3_/Q_4_ ratio was 1.12, 1.11, 1.09, and 1.13 for ILS-Ox3-40, ILS-Ox4-40, ILS-K3-40, and ILS-K4-40, respectively), which resulted in the partial condensation and formation of the silanol groups. The ^29^Si MAS NMR spectra also showed that the observed peaks were broad. This indicated that the obtained materials had a locally disordered silica skeleton, which was also observed for the unmodified mesoporous silica. The incorporation of the propylpyridinium groups into the silica structure was also confirmed by ^13^C MAS NMR analysis. It indicated that the signals corresponded to C=C and C=N of the pyridine ring and signals of hydrocarbon chain. Peaks at 42.1 and 42.0 ppm corresponded to CH_2_-C=O (samples’ series ILS-K3 and ILS-K4, respectively), peaks at 51.0 and 51.2 ppm corresponded to CH_2_-C=N (samples’ series ILS-Ox3 and ILS-Ox4, respectively), and peaks in the range of 32.1–7.5 ppm were due to the methyl and methylene carbons (CH_2_, CH_3_ and CH_2_-Si). It did not indicate the presence of the CH_3_-O group linked to the Si atom.

FTIR analysis confirmed the successful synthesis of the ionic liquid-functionalized silica. It indicated the vibrational bands assigned to the silanol (3200–3600 cm^−1^) and siloxane Si-O-Si and Si-O-Si-C moieties (1000–1200 cm^−1^). The symmetric vibrations of the Si-O-Si species were also observed as a broad band at 798-800 cm^−1^. The incorporation resulted in the absorption bands at 2992–2861 cm^−1^ assigned to the C–H asymmetric and symmetrical stretching vibrations and bands corresponding to the stretching C=C and C=N bonds of the pyridine ring (1440–1470 and 1631–1638 cm^−1^, respectively). The signal corresponding to the C=O group, confirming the incorporation of the pyridinium moiety with the ketone substituent, appeared at 1701 and 1699 cm^−1^ for the samples’ series ILS-K3 and ILS-K4, respectively. In the case of the functionalities consisting of a substituent of the oxime group, the bands corresponding to oxime C=N appeared at 1648 and 1652 cm^−1^ (for samples’ series ILS-Ox3 and ILS-Ox4, respectively). In the 1600–1680-cm^−1^ region, apart from the pyridine and oxime C=N bands, there may have been vibrational bending of the H-OH bond, resulting in a significant bandwidth broadening.

Based on elemental analyses, the amounts of the corresponding propylpyridinium groups were calculated and the results are presented in Table 1. The calculations performed showed that the involvement of the functional groups in the silica structure did not differ significantly from the assumed values (4–10%). However, more effective functionalization can be observed for moieties bearing the ketone or oxime substituent at the 4-position of the pyridine ring than in the case of those having the substituent at the 3-position. Probably, the location of the substituent at the 3-position caused a steric hindrance, limiting the concentration of the functionalities in the silica structure.

#### 3.1.2. Surface Morphology

**Figure 3:**
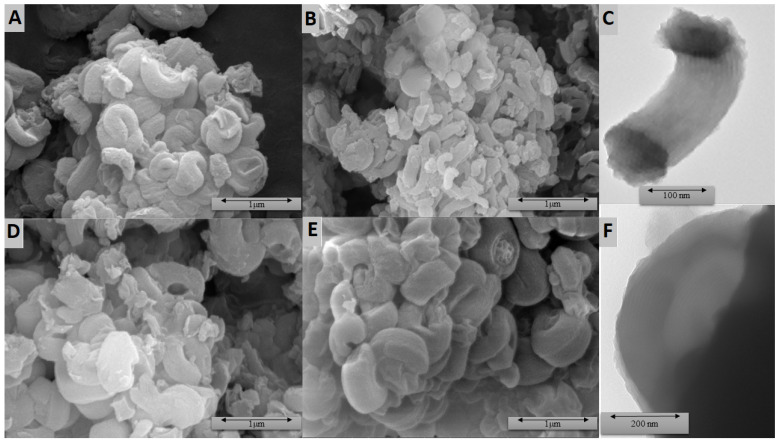
SEM (**A**,**B**,**D**,**E**) and TEM (**C**,**F**) micrographs of ILS-Ox3-20 (**A**), ILS-Ox4-20 (**B**,**C**), ILS-K3-20 (**D**), and ILS-K4-20 (**E**,**F**).

From the SEM micrographs of the selected fabricated materials, it was seen that, using the synthesis procedure, the highly ordered particles were obtained (Figure 3). However, the shape of the particles significantly depended on the structure and quantity of the silyl group. For the samples’ series ILS-Ox4, highly ordered, elongated structures were clearly observed at each concentration of the functional groups. They made up the majority of the sample material (round grains were also observed), and that pointed to significant regularity of the surface. It was also observed that the elongated grains were 100 to 500 nm wide, while the length was several hundred to 1000 nm, and they formed mainly a layered arrangement. This structure is typical for SBA-15 with uniform hexagonal pores. The rod-shaped SBA-15 particles also had a specific size: a width of 300–500 nm and a length of 700–1000 nm. For the series ILS-Ox3, elongated, buckled grains were observed for particles fabricated using the 40-fold excess of TEOS to the sililpropylpyridinium reagent (ILS-Ox3-40). They were about 300 nm wide and up to 1500 nm long. For the sample ILS-Ox3-20, round grains with a size of about 200–500 nm dominated. ILS-Ox3-10 contained large, multi-micron grains of irregular character and terraced structure. The elongated structures were visible for the ILS-K3-40 sample, but did not constitute the majority of the sample. Their widths were 100–200 nm and lengths were up to 1000 nm. For ILS-K3-20, a platelet shape dominated, and the particles were arranged in layers. The grain sizes were varied and ranged from below 100 to 1000 nm. For ILS-K3-10, medium and large platelet shapes were formed with a size of up to a few micrometers, also forming a terraced structure. In the case of the ILS-K4 series, the characteristic structures appeared most clearly for the sample ILS-K4-20. They had the form of coiled strips with a thickness of about 200 nm. Only individual grains were not strongly folded or formed into circles. For ILS-K4-40, a lot of irregular grains with a size of about 200–500 nm were observed. Only a small amount was elongated. For ILS-K4-10, large grains were observed, resulting from a combination of smaller ones. Some of them contained elongated structures. Figure 3 also shows the TEM images, which clearly reveal the parallel or vertical orientation of channels along the long axis, indicating that the resulting materials had an ordered, two-dimensional mesostructure of the channels.

#### 3.1.3. Textural Properties

**Figure 4:**
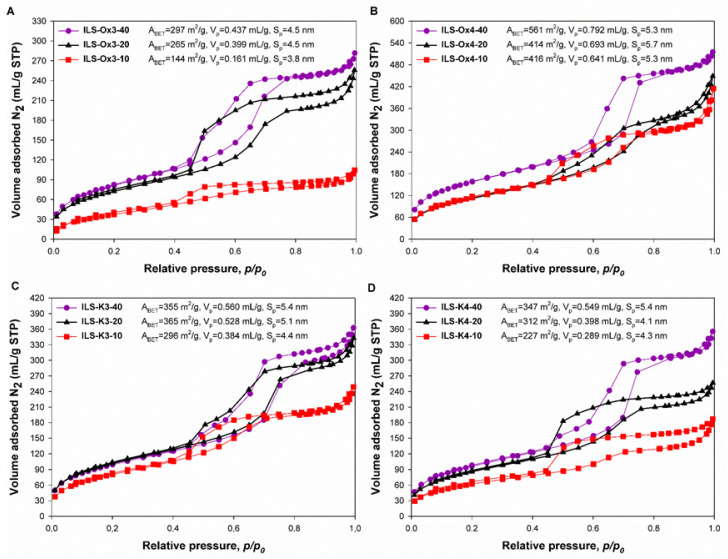
N_2_ adsorption/desorption isotherms of (**A**) ILS-Ox3, (**B**) ILS-Ox4, (**C**) ILS-K3, and (**D**) ILS-K4 materials.

It is well known that textural properties of the obtained materials have a crucial impact on their adsorption activity. In view of the above, to evaluate the textural properties for all prepared products, low-temperature N_2_ sorption was applied. As can be observed in Figure 4, the N_2_ adsorption/desorption isotherms of all analyzed materials were typical for type IVa according to the IUPAC classification, which is characteristic for mesoporous structures. Moreover, for all fabricated products, the H2 hysteresis loop, with cage-like structures and/or constricted mesopores, was observed. Interpretation of the obtained results proved that ILS-Ox4-40, ILS-Ox4-20, and ILS-Ox4-10 samples were characterized by the highest value of A_BET_ among all of the fabricated products. The values of BET surface areas for the mentioned samples were equal to 561 m^2^/g, 414 m^2^/g, and 416 m^2^/g of ILS-Ox4-40, ILS-Ox4-20, and ILS-Ox4-10 samples, respectively. The total pore volume (V_p_) for ILS-Ox4-40 sample was 0.792 mL/g, while the total pore volumes of ILS-Ox4-20 and ILS-Ox4-10 samples decreased to values of 0.693 mL/g and 0.641 mL/g, respectively. The average pore diameters (S_p_) of the analyzed products were 5.3 nm (ILS-Ox4-40 sample), 5.7 nm (ILS-Ox4-20 sample), and 5.3 nm (ILS-Ox4-10 sample). According to the obtained results for the parameters of the porous structure of the fabricated products, it was noted that the ILS-Ox3-40, ILS-Ox3-20, and ILS-Ox3-10 samples were characterized with the lowest BET surface area among all the synthesized materials. For the mentioned samples, the values of A_BET_ were 297 m^2^/g, 265 m^2^/g, and 144 m^2^/g for the ILS-Ox3-40, ILS-Ox3-20, and ILS-Ox3-10 samples, respectively. The average pore diameters of these products were 4.5 nm (ILS-Ox3-40 and ILS-Ox3-20 samples) and 3.8 nm (ILS-Ox3-10 sample), while the values of the total pore volume were 0.437 mL/g (ILS-Ox3-40 sample), 0.399 mL/g (ILS-Ox3-20 sample), and 0.161 mL/g (ILS-Ox3-10 sample), respectively. In the case of all fabricated adsorbents, it was noted that their synthesis had significant influence on the parameters of the porous structure. According to the obtained results, it was observed that the BET surface area of the fabricated products decreased as the addition of the organic functionalities increased. Moreover, the increase of the functionalities’ addition was also accompanied by the decrease in the volume of pores.

#### 3.1.4. Results of XRD Analysis

The XRD patterns of the fabricated ionic liquid silicas showed an intense diffraction peak in the range of 0.82–0.92° (indexed as (100)) and two low-intensity peaks (indexed as (110) and (200)) in the range of 1.44–1.79°, which are characteristic for mesoporous materials with highly ordered and textural uniformity (e.g., cubic (lm3m) or hexagonal (p6mm) symmetry [40,41,42]). Although the morphology analysis showed that the size and shape of the particles differed depending on the type of functionalizing group and its amount in relation to TEOS, the significant differences in the symmetry and the XRD study did not show the significant differences in the symmetry (Table 2).

In the case of the materials’ series ILS-Ox3, the peak (100) reflected a *d* spacing of 10.1 nm for ILS-Ox3-10 and 10.2 nm for ILS-Ox3-20 and ILS-Ox3-40, corresponding to the unit cell parameter (a_o_) 11.7, 11.7, and 11.8 nm, respectively. The location of the oxime substituent at the 4-position of the pyridine ring resulted in the lower *d* (100) spacing (9.9, 10.0, and 9.6 nm for ILS-Ox4-10, ILS-Ox4-20, and ILS-Ox4-40, respectively) and correspondingly lower values of the unit cell parameters (11.4, 11.5, and 11.6 nm), which may have been due to the dense location of the propyl pyridinium moieties. A similar effect was observed for the ILS-K3 and ILS-K4 series, but the differences were more visible. For the series ILS-K3, the peak (100) reflected a *d* spacing of 9.6, 10.1, and 10.7 nm for ILS-K3-10, ILS-K3-20, and ILS-K3-40, respectively, and these results corresponded to a_o_ = 11.1, 11.7, and 12.3 nm, respectively.

For the series ILS-K4, as expected, *d*-values were indicated as 9.7, 9.8, and 9.9 nm for ILS-K4-10, ILS-K4-20, and ILS-K4-40, respectively, and those resulted in values of the unit cell parameters 11.2, 11.3, and 11.4 nm, respectively. It was also indicated that the diffraction peak (100) intensities decreased as the TEOS excess decreased. It seemed that the cause of this effect was not the loss of crystallinity but the increasing number of organic groups, which was also observed in other functionalized silicas [43]. From the values of a_o_ and the pore width determined by the BJH method, the wall thicknesses were calculated. The walls obtained were generally smaller for materials containing more functional groups in their structure than in the case of materials obtained with the 40-fold excess of TEOS.

### 3.2. Sorption Studies

#### 3.2.1. Effect of pH

**Figure 5:**
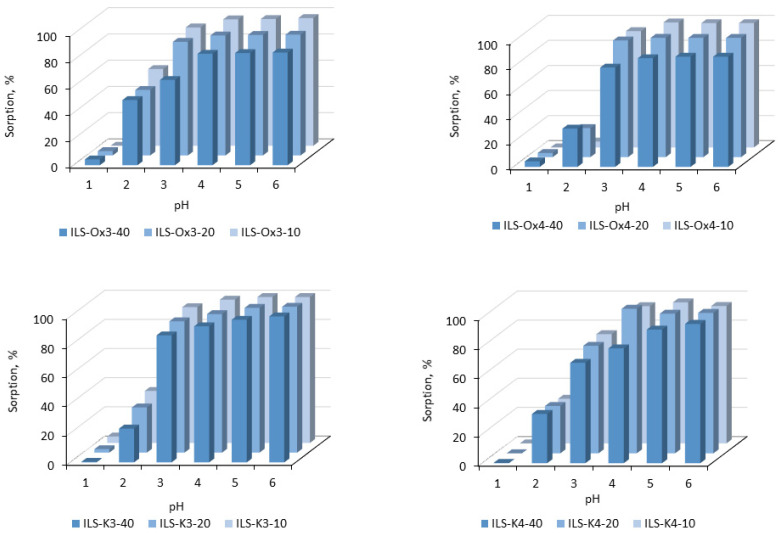
Effect of pH on Pb(II) adsorption onto the fabricated ionic liquid-functionalized silicas ([Pb((II) = 50 mg/L; pH = 6; sorbent dosage, 0.1 g; V, 100 mL).

The pH of the aqueous solution from which Pb(II) ions were removed significantly affected the adsorption process, both through the speciation of the metal ions and the surface charge of the sorption material. Given the differences in the content and structure of the functional groups and the morphology of individual series of the functionalized silicas, the effect of pH was analyzed for each ILS samples. The results obtained are presented in Figure 5. The data obtained showed that, regardless of the type of sorbents, the sorption almost did not occur when the pH was 1, and the further increase in the pH value increased the efficiency of the sorption. A rapid increase in the sorption efficiency was noted up to pH 4; above this value the amount of the metal ions removed remained constant (100% removal). Additionally, in the region of the increase in the sorption efficiency, the effect of the composition and morphology of the sorbent tested on the dynamic of the changes was observed. The most effective sorption at pH 2 was observed for the ILS-Ox3 series, and the amount of the metal ions removed increased with the increase in the amount of the functional group in the silica structure. A positive effect of the presence of the functional group on the Pb(II) removal was also observed at pH 3, but with higher efficiency than that at pH 2. The comparable dependencies were observed for the ILS-K3 series, except that sorption at pH 2 did not exceed 35% and at pH 3 it reached almost 100%. In the case of the ILS-K4 series, the positive effect of the presence of the functional group on the Pb(II) removal was observed at a pH of 4. For the ILS-Ox4 series, increasing the amount of the functional groups in the sorbent structure decreased the sorption to 11%, which made it possible to use much milder desorption conditions than in the case of other materials.

The XPS analysis of the functionalized silicas before and after the adsorption also confirmed the interaction of the functional group with the metals’ cations [44,45,46]. For the series ILS-K3 and ILS-K4, before adsorption the high-resolution C 1s spectra were deconstructed into peaks at 284.8, 286.4, and 289.8, which were assigned to C=C, C=O, and C-O, respectively. The N1s spectra was resolved into two component signals attributed to aromatic C=N at 399.4 eV and pyridinium-N^+^- at 402.1 eV species [47]. The O 1s spectra showed only a single line centered at 532.6 eV, which indicated the presence of C-O bonds. After the adsorption, the N1s spectra showed that the aromatic C=N was shifted to 399.6–400.1 eV, while the pyridinium-N^+^- was shifted to 401.6–401.8 eV species. In the case of the ILS-Ox3 and ILS-Ox4 series, the O 1s spectra showed that the signal observed at 532.6 eV was shifted to 533.1 eV and signals attributed to aromatic and imine C=N (399.4 eV) as well as pyridinium-N^+^- (402.1) eV were shifted to 400.4 eV. The obtained results confirmed that ketone as well as oxime substituents reacted with the metals’ species during the process of adsorption.

#### 3.2.2. Adsorption Kinetics

**Figure 6:**
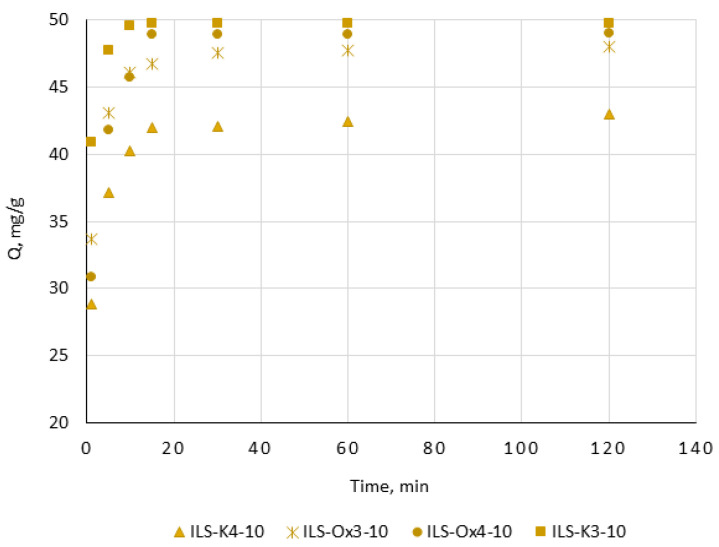
Adsorption kinetics on the selected ionic liquid-functionalized silica ([Pb((II) = 50 mg/L; pH = 6; sorbent dosage, 0.1 g; V, 100 mL).

Another parameter describing the solute sorption onto a sorbent surface is the kinetics of adsorption. It provides knowledge not only about the effectiveness, but also mechanism of the process. The tests were conducted from 1 to 120 min, at a temperature of 23 °C, and using all fabricated materials. The aqueous solution used for the tests consisted of Pb(II) in a concentration of 50 mg/L, and pH was 6. The most representative results plotted as the amount of Pb(II) adsorbed as a function of the contact time are shown on Figure 6. It can be observed that, in the case of silica functionalized with 1-[3-(trimethoxysilyl)propyl]-3-undecanoylpyridinium bromide (ILS-K3-10), the adsorption was rapid in the first 5 min, but the maximum adsorption was reached after 10 min of the contact time. In the case of ILS-K4-10, sorption also increased rapidly in the first 5 min, but a further course of the adsorption indicated that the equilibrium was achieved slowly. ILS-Ox3-10 showed slightly higher sorption dynamics than ILS-K4-10, while ILS-Ox4-10 achieved the maximum sorption in the 15th minute of the process.

The experimental data were fitted to the nonlinear forms of the kinetic models: pseudo-first order, pseudo-second order, Elovich, and intra-particle diffusion model [48,49,50,51]. The results of the fitting showed that, irrespective of the structure of the functional group, the pseudo-second-order model provided the best fit to the experimental data. The values of the determination coefficients (R^2^) estimated from the adjustment were 0.997, 0.993 0.998, and 0.994 for ILS-K3-10, ILS-K4-10, ILS-Ox3-10, and ILS-Ox4-10, respectively. These results pointed Pb(II) interacting with the sorbent surface through chemical reaction, e.g., interaction of the electrophilic Pb^2+^ with the nucleophilic substituent in the pyridine ring. Moreover, the estimated kinetic constants confirmed that sorption of Pb(II) onto ILS-K3-10 was much faster (0.059 g/mg·min) with respect to ILS-K4-10, ILS-Ox3-10, and ILS-Ox4-10 (0.039, 0.038, and 0.053 g/mg·min, respectively).

#### 3.2.3. Adsorption Isotherms

**Figure 7:**
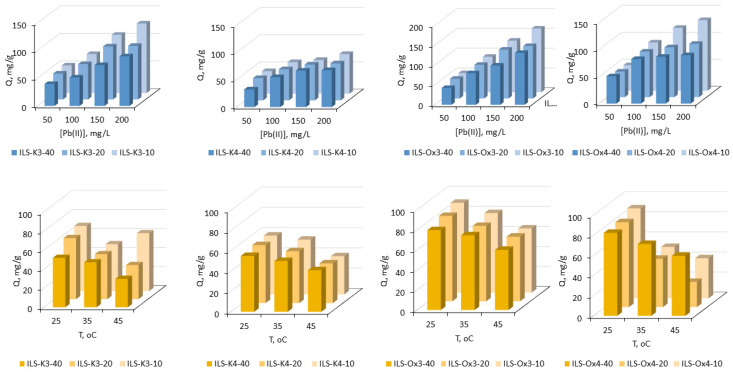
Effect of initial Pb(II) concentration and temperature on the removal of Pb(II) using IL-functionalized silica (time = 120 min; pH = 6; sorbent dosage, 0.1 g; V, 100 mL; [Pb(II)] during the temperature test = 100 mg/L).

The initial concentration of solute had a large influence on the removal and capacity of the sorbent; therefore, the effect of the initial Pb(II) concentration on sorption efficiency was also studied. The test was evaluated using the initial Pb(II) concentration ranging from 50 to 200 mg/L, at a constant temperature (23 °C), shaking time (120 min, which was determined to be enough to achieve the equilibrium state), and at pH of 6. As shown in Figure 7, for all tested materials, the amount of adsorbed Pb(II) increased when the initial Pb(II) concentration increased. These results also showed that not all materials obtained revealed significant adsorption properties. A slight increase in the *q* value was observed when the materials’ series ILS-K4 were used. In this case, regardless of the amount of the functional groups incorporated into the silica structure, at a concentration of 200 mg/L Pb(II), the parameter *q* did not exceed 73 mg/g Pb(II). Significantly better values of *q* were observed for the materials’ series ILS-K3 and ILS-Ox4. Both at the minimal and maximal participation of the functional groups in the silica structure, the amount of adsorbed Pb(II) ions increased with the increase of the initial Pb(II) concentration. However, the number of the functional groups significantly affected the efficiency of the process, and ILS-K3-10 and ILS-Ox4-10 showed the highest adsorption capacity (126.1 and 130.7 mg/g, respectively). The most efficient series turned out to be ILS-Ox3; ILS-Ox3-10 removed not less than 80% of the metal ions (q = 161 mg/g after contact with 200 mg/L of Pb(II)).

Despite the clear dependence of the sorption efficiency on the number of functional groups in the silica structure, the 40-fold excess of TEOS in the synthesis of ILS-Ox3 and ILS-K4 resulted in more promising materials than using the 10-fold excess. This was indicated by Langmuir, Freundlich, Dubinin–Radushkevich, and Temkin isotherm models, selected from the most commonly employed models to describe the adsorption process in heterogeneous systems [52,53]. The sorption parameters of the selected isotherms models, obtained by non-linear regression analysis, are summarized in Table 3. In both cases, the Langmuir model provided a better fit to the experimental data than the other considered models (R^2^ = 0.996−0.999). These results pointed to homogenous adsorption by forming a self-assembled monolayer of the adsorbate. Moreover, the values of parameter R_L_ (separation factor) were in the range of 0–1, indicting the adsorption as favorable, while the capacity of each sample was 171.9, 141.6, and 163.3 mg/g for ILS-Ox3-40, ILS-Ox3-20, and ILS-Ox3-10, respectively, and 85.2, 72.3, and 78.0 mg/g for ILS-K4-40, ILS-K4-20, and ILS-K4-10, respectively.

In the case of the ILS-K3 series, the adsorption equilibrium followed both Langmuir and Freundlich models. However, the Langmuir model showed a significantly better fit for the sample ILS-K3-40. However, for ILS-K3-10, the best fitting was obtained by the Freundlich model, according to which the adsorption occurred on a heterogeneous surface with non-uniform distribution of active sites. Possibly, a dense arrangement of the functional groups limited access to the silanol groups, which resulted in Pb(II) coordination mainly through pyridinium moieties. However, in the case of less participation of the functional group in the silica surface, the Pb(II) species had both the possibility of interaction with the silica matrix and with the pyridinium groups. The Langmuir model also served to estimate the maximum adsorption capacities, and the *Q_m_* values using ILS-Ox3-40, ILS-Ox3-20, and ILS-Ox3-10 were 171.9, 141.6, and 163.3 mg/g, respectively. For the ILS-Ox4 series, the Freundlich model provided a satisfactory fit with *R^2^* values close to 1.000, indicating heterogeneity of the surface. Moreover, the values of the Freundlich constants (K_F_ = 61.5, 62.3 and 49.2) and values of n (11.2, 10.4, and 4.1) for ILS-Ox4-40, ILS-Ox4-20, and ILS-Ox4-10, respectively, indicated strong chemical interaction between the solid phase and Pb(II).

The effect of temperature on the adsorption equilibrium was also examined using the aqueous solutions containing 100 mg/L Pb(II). The results obtained are presented in Figure 7. As can be seen, for all fabricated IL-functionalized silicas, the adsorption of Pb(II) was less efficient as the temperature increased from 25 to 35°C. For the series ILS-K3, the decrease increased as the amount of the functional group in the silica increased (9.1, 26.3, and 28.0% for ILS-K3-40, ILS-K3-20, and ILS-K3-10, respectively). For the series ILS-Ox3 and ILS-Ox4, the lowest decrease was observed for the materials consisting of the lowest amount of the functional groups (for ILS-Ox3-40, decrease of 6.5%; for ILS-Ox4-40, decrease of 13.4%). For ILS-Ox3-20 and ILS-Ox3-40, as well as for ILS-Ox4-20 and ILS-Ox4-10, the decreases were comparable (11.5 and 11.7%, as well as 43.0 and 42.9%, respectively). In the case of the series ILS-K4, the above-mentioned relationships were not recorded (decrease in 9.0, 10.5, and 7.0%, respectively). A further increase in temperature for the series ILS-K4, ILS-Ox3, and ILS-Ox4, as well as for ILS-K3-40 and ILS-K3-20, made the adsorption even less efficient, pointing to the exothermic nature of the processes. The calculated thermodynamic parameters (ΔH, ΔS, and ΔG) also confirmed this conclusion [54]. The values of ΔH< 0 confirmed the nature of adsorption (for the series ILS-K4, ΔH = −43.0, −55.0, and −62.2 kJ/mol; for the series ILS-Ox3, ΔH = −73.4, −89.5, and −117.6 kJ/mol; and for ILS-Ox4, Δ = −88.0, −207.5, and −189.0 kJ/mol; and ΔH = −69.9 and −88.8 kJ/mol for ILS-K3-40 and ILS-K3-20, respectively). The negative values of ΔG° at 25 °C also indicated the adsorption as spontaneous, but negative values of ΔS (for the series ILS-K3 ΔS = −232.7, −293.2, and −84.7 J/mol K; for the series ILS-K4, ΔS −142.1, −181.4, and −205.3 J/mol K; for the series ILS-Ox3, ΔS = −234.1, −285.4, and −376.6 J/mol K; and for ILS-Ox4, ΔS = −282.4, −683.0, and −619.1 J/mol K, respectively) demonstrated the decreased randomness at the solid–solution interface during adsorption.

The estimated adsorption capacity of ILS-Ox3-40 was significantly higher than that of other functionalized silica-based adsorbents being potentially suitable for such applications (Table 4). The capacity was twice as high as the value obtained for *N*-propyl-2-pyridylimine-functionalized silica [55] and almost three times as high for thiol-functionalized MCM-41 [22].

### 3.3. Desorption Studies

Based on the obtained results, the various concentrations (0.01–1 M) of HCl, H_2_SO_4,_ and HNO_3_ were tested as desorbing agents for Pb(II) from ILS-K3-10, ILS-K4-10, ILS-Ox3-10, and ILS-Ox4-10. For the tests, the materials after sorption with 200 mg/L Pb(II) at pH of 6 were used. The experiments were conducted for 15 min, at a temperature of 25 °C, using 1 g of the loaded silicas and 100 mL of the desorbing agent. The results showed that 0.1 M solution of mineral acid gave efficient desorption (92–99%); however, treatment of the loaded ILS-K3-10, ILS-K4-10, and ILS-Ox4-10 with 0.01 M aqueous HCl resulted in less efficient desorption (Table 5). Moreover, the repetition of the sorption–desorption procedure without a washing stage also indicated that the selected materials can be reused after desorption without significant loss in their sorption properties (±5%). After desorption, the infrared spectra of the centrifuged and dried sorbents were compared with the spectra of the sorbents before the Pb(II) removal. The comparison showed no significant deviations in the position and intensity of the bands corresponding to the vibrations of the pyridinium ring (C=C at 1440–1470 cm^−1^ and C=N at 1631–1638 cm^−1^), as well as the bands corresponding to the oxime C=N (at 1649 and 1652 cm^−1^ for LS-Ox3-10 and ILS-Ox4-10, respectively) and ketone C=O groups (at 1702 and 1698 cm^−1^ for ILS-K3-10 and ILS-K4-10, respectively). These results confirmed the stability of the functionalization, especially in contact with the acidic desorbing agent.

### 3.4. Adsorption from Wastewater

Adsorption performance of the selected sorbent was evaluated by the experiments carried out using a synthetic gold mining effluent containing 85.00 mg/L Pb(II), 2.59 mg/L Cu(II), and 0.42 mg/L Zn(II) [62]. In this test, 100 mg of ILS-Ox3-40 was dispersed into 100 mL of the aqueous solution, and the mixture was shaken for 15 min. It was found from the obtained results that Pb(II) was removed by 99.2% (q = 84.31 mg/g), Cu(II) by 0.8% (q = 0.03 mg/g), and Zn(II) by 4.7% (q = 0.02 mg/g). These results indicated the high potential of ILS-Ox3-40 for the treatment of wastewater containing mainly Pb(II).

## 4. Conclusions

In this study, novel ionic liquid-functionalized silicas were synthesized through co-condensation at different molar ratios of TEOS to the ionic liquid group precursor (1-[3-(trimethoxysilyl)propyl]-3-undecanoylpyridinium bromide, 1-[3-(trimethoxysilyl)propyl]-4-undecanoylpyridinium bromide, 3-[1-(hydroxyimine)undecyl]-1-[3-(trimethoxysilyl)propyl]pyridinium bromide, and 4-[1-(hydroxyimine)undecyl]-1-[3-(trimethoxysilyl)propyl]pyridinium bromide). Moreover, the conducted analyses (NMR, FT-IR, and elemental) confirmed the incorporation of the functional groups and that their involvement in the silica structure did not differ significantly from the assumed parameters. The SEM and TEM micrographs of the fabricated materials indicted that, using the synthesis procedure, highly ordered particles with a two-dimensional mesostructure were obtained. These results were also in agreement with the XRD patterns, showing peaks characteristic for mesoporous materials with a highly ordered and textural uniformity. The fabricated materials were used as sorbents for the removal of Pb(II) from aqueous solutions. It was shown that almost all Pb(II) ions were removed from the solution with a pH above 3, which can be attributed to the fact that the Pb^+2^ species was the most abundant under these conditions and it was not displaced from the sorbent surface by H^+^, which took place in contact with the strongly acidic solution. Moreover, regardless of the type and amount of the functional groups incorporated into the silica structure, the kinetic of the sorption was fast (5 and 10 min) and the adsorption efficiency exceeded 70 mg/g. The most promising sorbent of Pb(II) was ILS-Ox3-40, with the estimated capacity of 172 mg/g. The studies also showed that for most samples the Langmuir model provided a better fit to the experimental data than the other considered models, which pointed to homogenous adsorption by forming a self-assembled monolayer of the adsorbate. However, in the cases of series ILS-Ox4, sample ILS-K3-40, and sample ILS-K3-10, the best fitting was obtained by the Freundlich model, according to which the adsorption occurred on a heterogeneous surface with a non-uniform distribution of active sites. For the fabricated silicas, decreased sorption with an increase in temperature was also observed, indicating the exothermic and spontaneous nature of the adsorptions. Other tests showed that adsorbed Pb(II) can be easily desorbed using a diluted acid solution, and the regenerated sorbent can be reused after desorption without loss in the sorption properties.

## Figures and Tables

**Table 1:** Results of elemental analysis of the fabricated functionalized silicas.

Sample	Si/N ^1^ (Theoretical)	C_f_^2^ mmol/g
ILS-Ox3-10	6.2 (5.5)	0.89 (0.96)
ILS-Ox3-20	11.4 (10.5)	0.57 (0.61)
ILS-Ox3-40	22.6 (20.5)	0.32 (0.35)
ILS-Ox4-10	5.9 (5.5)	0.91 (0.96)
ILS-Ox4-20	11.3 (10.5)	0.57 (0.61
ILS-Ox4-40	21.4 (20.5)	0.34 (0.35)
ILS-K3-10	12.9 (11.0)	0.87 (0.97)
ILS-K3-20	23.7 (21.0)	0.56 (0.61)
ILS-K3-40	43.6 (41.0)	0.33 (0.35)
ILS-K4-10	11.8 (11.0)	0.93 (0.97)
ILS-K4-20	23.1 (21.0)	0.57 (0.61)
ILS-K4-40	42.8 (41.0)	0.34 (0.35)

^1^ Si/N, molar ratio of the elements; ^2^ C_f_, the functional group concertation; both calculated from elemental analysis.

**Table 2:** Textural data of the ionic liquid-functionalized silicas and examples of XRD patterns.

	Sample	d (100) nm	d (110) nm	d (200) nm	a_o_^1^ nm	Wall Thickness ^2^ nm
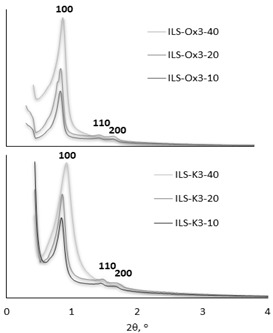	ILS-Ox3-10	10.1	5.8	5.1	11.7	7.6
	ILS-Ox3-20	10.2	5.9	5.1	11.7	6.3
	ILS-Ox3-40	10.2	5.9	5.1	11.8	6.7
	ILS-Ox4-10	9.9	5.8	5.0	11.4	5.7
	ILS-Ox4-20	10.0	5.8	5.0	11.5	5.5
	ILS-Ox4-40	10.1	5.9	5.0	11.6	6.3
	ILS-K3-10	9.6	6.0	5.0	11.1	6.4
	ILS-K3-20	10.1	6.1	5.2	11.7	6.2
	ILS-K3-40	10.7	6.1	5.4	12.3	6.4
	ILS-K4-10	9.7	5.8	4.9	11.2	6.3
	ILS-K4-20	9.8	5.8	5.0	11.3	6.2
	ILS-K4-40	9.9	5.8	5.1	11.4	5.6

^1^ Determined by the low-angle XRD (d_(100)_, interplanar distance plane (100); a_o_, distance between centers of two adjacent pores). ^2^ Calculated from the relation of a_o_/2 = d_BJH_ + t where d_BJH_ is pore width determined by the BJH method and *t* is wall thickness between adjacent pores.

**Table 3:** Isotherms’ parameters of different models for sorption of Pb(II) with the fabricated IL-functionalized silica.

Isotherm Model	ILS-K3			ILS-K4		
	ILS-K3-40	ILS-K3-20	ILS-K3-10	ILS-K4-40	ILS-K4-20	ILS-K4-10
**Langmuir**						
**Q_m_ (mg/g)**	104.6	99.7	127.4	85.2	72.3	78.0
**K_L_ (L/mg)**	0.05	0.15	0.34	0.04	0.12	0.10
**R^2^**	0.988	0.996	0.992	0.996	0.999	0.999
**Freundlich**						
**K_F_ (mg^1−(1/n)^/gL^n^)**	17.9	37.7	67.5	2.5	5.1	4.9
**n**	2.9	5.0	7.4	11.1	26.9	27.4
**R^2^**	1.000	0.998	0.997	0.939	0.988	0.994
***Dubinin*** **–** ***Radushkevich***						
**Q_m_(mg/g)**	78.0	85.4	107.5	67.5	64.2	67.0
**K_DR_ (mol^2^/kJ^2^)**	1.3 × 10^−5^	1.2 × 10^−6^	2.2 × 10^−8^	4.0 × 10^−5^	6.6 × 10^−6^	5.5 × 10^−6^
**R^2^**	0.859	0.884	0.884	0.971	0.912	0.867
**Temkin**						
**K_T_ (L/g)**	0.62	9.90	820.0	0.33	5.72	4.73
**B**	119.0	98.5	230.4	127.8	237.7	217.9
**R^2^**	0.985	0.982	0.961	0.969	0.981	0.988
	**ILS-Ox3**			**ILS-Ox4**		
	**ILS-Ox3-40**	**ILS-Ox3-20**	**ILS-Ox3-10**	**ILS-Ox4-40**	**ILS-Ox4-20**	**ILS-Ox4-10**
**Langmuir**						
**Q_m_ (mg/g)**	171.9	141.6	163.3	92.6	97.8	141.3
**K_L_ (L/mg)**	0.05	0.49	0.7	0.7	0.5	0.2
**R^2^**	0.999	0.997	0.996	0.944	0.811	0.823
**Freundlich**						
**K_F_ (mg^1−(1/n)^/gL^n^)**	17.2	71.8	80.2	61.5	62.3	49.2
**n**	2.0	6.2	5.2	11.2	10.4	4.1
**R^2^**	0.988	0.988	0.993	1.000	1.000	1.000
***Dubinin*** **–** ***Radushkevich***						
**Q_m_(mg/g)**	112.1	117.8	129.9	87.2	90.2	112.4
**K_DR_ (mol^2^/kJ^2^)**	9.0 × 10^−6^	2.4 × 10^−8^	2.7 × 10^−8^	1.6 × 10^−8^	1.7 × 10^−8^	2.8 × 10^−7^
**R^2^**	0.901	0.866	0.859	0.964	0.947	0.861
**Temkin**						
**K_T_ (L/g)**	0.42	301.4	138.7	3.8 × 10^4^	1.6 × 10^4^	10.1
**B**	64.0	180.7	137.4	410.2	370.8	123.0
**R^2^**	0.981	0.888	0.987	0.988	0.985	0.983

**Table 4:** Comparison of ILS-Ox3-40 with other adsorbents.

Adsorbent	Capacity mg/g	Reference
SH-functionalized MCM-41	66.0	[22]
Ethylenediamine-functionalized SBA-15	96.4	[56]
Shift base-functionalized SBA-15	60.9	[57]
	82.1	[55]
Chitosan-functionalized MCM-41	90.9	[58]
EDTA-modified chitosan/SiO_2_/Fe_3_O_4_	123.4	[59]
alumina-containing MCM-41	153.0	[60]
Fe_3_O_4_-CS-L	128.6	[61]
ILS-Ox3-40	171.9	This work

**Table 5:** Results of desorption tests.

Adsorbent	Desorption Agent HCl mol/L	Desorption %	Efficiency Loss ^1^ %
ILS-K3-10	0.01	87.7	3.8
	0.1	91.8	5.3
ILS-K4-10	0.01	82.6	4.7
	0.1	99.1	4.9
ILS-Ox3-10	0.01	60.7	5.1
	0.1	99.4	5.4
ILS-Ox4-10	0.01	97.2	4.7
	0.1	99.2	5.4

^1^ Efficiency loss calculated after five sorption–desorption cycles; composition of the aqueous solution used for sorption: 200 mg/L Pb(II), pH = 6.

## Data Availability

Not applicable.

## Sample Availability

Samples of all synthetized compounds are available from the authors.
